# Investigating the Machining Quality of Additively Manufactured Composite: Multi-Response Modeling and Evolutionary Optimization

**DOI:** 10.3390/mi17040444

**Published:** 2026-04-02

**Authors:** Anastasios Tzotzis, Dumitru Nedelcu, Simona-Nicoleta Mazurchevici, Panagiotis Kyratsis

**Affiliations:** 1 Department of Product and Systems Design Engineering, University of Western Macedonia, 50100 Kila Kozani, Greece; 2 Department of Manufacturing Engineering, “Gheorghe Asachi” Technical University, 700050 Iasi, Romania; dnedelcu@tcm.tuiasi.ro (D.N.); simona-nicoleta.mazurchevici@academic.tuiasi.ro (S.-N.M.)

**Keywords:** additive manufacturing, CFRP, dimensional error, machining, NSGA-II, PET-G, regression analysis, surface roughness

## Abstract

This study investigates the turning performance of additive-manufactured polymer-based composite, with particular emphasis on the resulting dimensional error (*DE*) and surface roughness *Ra*. Cutting speed, feed rate, and depth of cut were selected as continuous process variables. Subsequently, regression-based modeling was applied to the experimental data, resulting in predictive models with a coefficient of determination (R^2^) equal to 96.35% and 92.88% for the *DE* and *Ra*, respectively. The analysis indicated that depth of cut and cutting speed accounted for more than 86% of the *DE* model’s explanatory power, while cutting speed, feed and depth of cut contributed approximately 90% to the *Ra* model. To further evaluate process performance, the Non-Dominated Sorting Genetic Algorithm II (NSGA-II) was employed to determine the Pareto-optimal solutions that simultaneously minimize the dimensional error and the surface roughness. It was found that the optimal solutions are generated with a cutting speed between 120 m/min and 180 m/min, depth of cut below 0.52 mm and feed ranging from 0.05 mm/rev to 0.10 mm/rev. Finally, additional validation experiments confirmed the reliability of the proposed models, yielding mean absolute prediction errors between the measured and estimated values equal to 3% for the dimensional error and 4.8% for the surface roughness.

## 1. Introduction

Surface integrity in machining plays a decisive role in the functional performance, appearance, and reliability of manufactured components. It is characterized by attributes such as surface roughness, waviness, and dimensional accuracy, which together define the topography quality and geometric precision of the finished surface. Among these characteristics, the average surface roughness (*Ra*) [1] is of particular importance, as it directly affects fatigue life, wear behavior, frictional response, and the adhesion performance of functional coatings. At the same time, dimensional error represents a critical quality indicator, reflecting the deviation of the machined geometry from its nominal dimensions and directly influencing assembly accuracy, interchangeability, and overall product reliability. Achieving both low surface roughness and minimal dimensional error is essential in manufacturing sectors involving high-precision components, such as the automotive, structural, and consumer sectors. These quality outcomes are strongly governed by machining parameters, including cutting speed, feed rate, depth of cut, and tool geometry. Furthermore, material characteristics, tool wear progression, and machine-tool vibrations may introduce additional variability, rendering the simultaneous control of surface finish and dimensional accuracy a complex and interdependent problem.

A high number of studies that are available In the literature deal with Investigations on the surface roughness and surface quality of a wide range of material groups under various manufacturing processes. Ramasamy et al. [2] investigated the machinability responsiveness of aluminum hybrid metal matrix under milling processes, in terms of the surface roughness. Dambatta et al. [3] evaluated the surface quality of Al6061-T6 during machining by utilizing nanofluid cooling. The authors focused on the developed cutting forces and surface roughness. Regarding steel alloys, Swain et al. [4] analyzed the relationship between surface roughness, tool wear and vibration during the high-speed dry machining of 1040 steel with uncoated tools. Vasanth et al. [5] proposed a fusion of cutting force, tool wear and tool vibration with respect to the cutting speed, feed and depth of cut, in order to predict the surface roughness of hardened SS 410 steel using multicoated cutting inserts. Soori et al. [6] worked on the minimization of surface roughness during the abrasive waterjet machining of titanium alloy Ti6Al4V. The study employed virtual environments based on the smoothed particle hydrodynamics approach and finite element method. Chauhan et al. [7] studied the characterization and classification of variations in surface roughness during the face milling of INCONEL 625 by utilizing cutting force signals. Their study investigated the influence of machining parameters on cutting forces and surface roughness as well. Finally, composite materials have also been studied. Duboust et al. [8] developed novel measurement methods involving optical surface analysis and image segmentation for calculating area surface roughness parameters of machined carbon-fiber composite laminate. In addition, statistical analysis was carried out and empirical models have been developed to predict surface roughness. Similarly, Kamath et al. [9] worked on the drilling of glass fiber reinforced polymers. The authors investigated the effect of tool geometry along with speed and feed on the delamination damage and surface roughness of the drilled holes.

Composites are continuously gaining ground in the manufacturing sector due to their increased stiffness, resistance and low weight. Especially with the advent of new 3D printing technologies and composite filaments, the manufacturing of 3D-printed components based on composite materials has significantly increased. Even though a high number of studies related to additive manufacturing (AM) are available in the bibliography, the post-processing of additive-manufactured parts and components is still understudied, especially of 3D-printed composites. Post-processing includes several techniques, mostly chemical such as vapor deposition and vat-photopolymerization post-curing [10,11,12]. Machining is another post-processing method for 3D-printed parts—a mechanical one, that in contrast to chemical methods, is based on material removal under certain parameters. Typically, such methods involve more complex and time-consuming setups; however, they lead to smoother and more precise surfaces. Moreover, they can be applied to virtually all 3D-printed materials, in contrast to chemical methods. Venkatesh et al. [13] studied the machinability possibilities of both additive-manufactured Polylactic Acid (PLA) and Polyethylene Terephthalate Glycol (PET-G) polymers under dry conditions with a CNC lathe. Cococcetta et al. investigated the milling possibilities of 3D-printed carbon fiber reinforced polymers (CFRP) under cryogenic conditions, with an aim to evaluate the surface integrity. Similarly, Das et al. [14] worked on the post-processing of polymer composites during milling with coated carbide tools. The authors focused on the developed cutting forces. Wang et al. [15] worked on material removal rate (MRR) optimization in robotic polishing by utilizing Bayesian algorithms. Another study related to wafer polishing [16] used deep learning-based techniques to model the relationship between process parameters and MRR.

A review of the existing literature Indicates that machining studies on additively manufactured materials remain relatively scarce, particularly for polymer-based composites. Moreover, limited attention has been given to the post-processing of 3D-printed CFRP components via conventional machining operations such as turning. The present work examines the effect of key machining parameters, namely cutting speed, feed rate, and depth of cut, on both surface roughness and dimensional error during the turning of 3D-printed CFRP. In contrast to the previous study of the authors [17], which focused on data-driven modeling and surface defect characterization, the present study adopts a regression-based framework to provide an interpretable and statistically grounded analysis of the machining process, in this way enabling the mechanistic understanding of the process. Particular emphasis is given on the combined evaluation and trade-off between surface quality and dimensional error (*DE*), which is critical for finishing operations in additively manufactured composites, especially in the absence of dedicated cutting tools. To this end, regression models are integrated with the Non-Dominated Sorting Genetic Algorithm II (NSGA-II) to identify optimal cutting conditions, while additional validation through PRESS and k-fold cross-validation is employed to ensure model robustness and predictive reliability.

## 2. Materials and Methods

**Figure 1:**
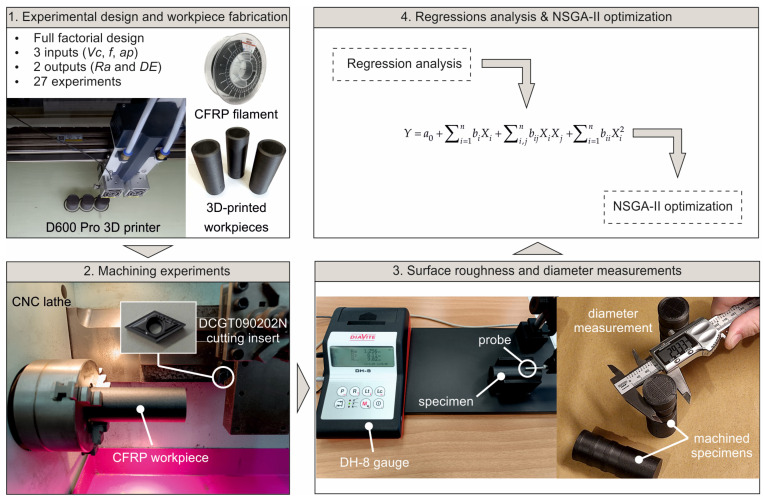
The experimental framework of the study.

This work focuses on the experimental evaluation of 3D-printed polymer-based composite during turning operations. The adopted methodology comprises four sequential stages, as illustrated in Figure 1. Initially, the experimental plan was formulated based on the selected machining conditions, resulting in a total of 27 cutting trials. Concurrently, the corresponding workpieces were manufactured with the aid of a CreatBot D600 Pro printer (Henan Creatbot Technology Limited, Zhengzhou City, China). Subsequently, the turning tests were performed using a 0.5 kW BOXFORD 160TCL CNC machine (Boxford Holdings Ltd., Halifax, UK), while the specimens were clamped with the self-centering three-jaw chuck. The machine has predefined X and Z datums, whereas tool offsets were defined according to the BOXFORD 160TCL (Boxford Holdings Ltd., Halifax, UK) setup procedure. The tool was mounted and the tool offset was established through a trial cut and subsequent diameter measurement, with corrections applied to the X-offset to approximate the nominal dimension. Calibration was performed prior to the experimental runs and was repeated on each insert replacement. Sumitomo DCGT090202N 55° diamond-shaped cutting inserts (SUMITOMO ELECTRIC Hartmetall GmbH, Willich, Germany) were used, with a 0.2 mm corner radius and 93° lead angle. In the third stage, surface roughness measurements and the diameter were acquired employing a DIAVITE DH-8 surface roughness tester (Diavite AG, Bülach, Switzerland), with a diamond stylus tip radius of 5 μm and cutoff length (λc) of 0.8 mm, and a digital vernier caliper with 0.01 mm resolution respectively. The surface roughness measurements were carried out with the ISO 21920-3 [18] guidelines based on the expected roughness magnitude, and the final values were calculated as the average of four measurements, taken at equivalent anti-diametral points of each machined circumference. Finally, the experimental results were systematically organized and analyzed through regression-based modeling with the use of MATLAB™ R2021.

### 2.1. Fabrication Settings and Material Specifications

Table 1 contains the composition of the polymer-based composite material used to fabricate the workpieces, as well as the specifications of the material as given by the manufacturer [19]. Take note that the elastic modulus was tested at 1 mm/min speed, whereas the yield strength, strain and strain at break were tested at 50 mm/min speed. This material was chosen for its superior properties compared to standard polymer filaments. The cylindrical workpieces have 30 mm diameter and 70 mm length. All specimens were fabricated in a single batch to ensure consistent processing conditions and to minimize variability associated with the large build platform (600mm × 600 mm× 600 mm). This approach reduces the influence of thermal gradients, bed-level inconsistencies, and machine reinitialization between prints. The specimens were positioned within a controlled region of the build plate to ensure uniform quality.

Table 2 summarizes the process parameters employed for the fabrication of the test specimens operating under the Fused Deposition Modeling (FDM) technique. A nozzle with a diameter of 0.4 mm was used in conjunction with 1.75 mm filament. All primary printing parameters were selected in accordance with the manufacturer’s guidelines. Particular attention was given to the selection of shell thickness (3 mm), taking into consideration the applied depth of cut during machining as well as the required structural stiffness of the specimens. Each specimen was produced with fully solid external walls of sufficient thickness to ensure continuous material engagement during turning, thereby preventing the cutting tool from encountering internal voids or discontinuities between adjacent deposited layers. Previous work [20] has shown that a layer thickness of 0.2 mm combined with a printing speed of 40 mm/s enhances interlayer bonding and material alignment. Accordingly, the layer height, printing speed, and filament flow rate were selected based on these findings to promote improved material cohesion. Finally, an infill density of 50% with a rectilinear pattern was adopted to provide adequate mechanical stability and secure clamping of the workpieces during the turning process.

Finally, Table 3 lists the machining parameters considered in the present investigation, namely cutting speed (*Vc*), feed rate (*f*), and depth of cut (*ap*), together with the corresponding ranges examined. The selected parameter intervals were defined based on the cutting tool manufacturer’s recommendations for light and finishing turning operations, supplemented by findings reported for the machining of unreinforced PET-G [13]. The cutting tests were carried out using DCGT090202N inserts mounted on an SDJCL1010 tool holder.

### 2.2. Design of Experiments for the Machining Tests

A full factorial experimental design was adopted in this study to systematically investigate the individual and interactive effects of the cutting speed, feed rate, and depth of cut on the machining responses. This approach enables the independent estimation of the main effects as well as two-factor and higher-order interactions, which is particularly important in machining processes where strong parameter coupling is commonly observed [21,22]. Given the limited number of control factors and the selected discrete levels, the full factorial design provides a comprehensive and statistically robust description of the process behavior without requiring an excessive number of experiments. Moreover, the complete coverage of the experimental space enhances the reliability of subsequent regression modeling and multi-objective optimization. Table 4 contains the test combinations, as well as the corresponding results. The experimental runs were performed in a fully randomized order to minimize systematic bias. All tests were conducted within a single session under stable environmental conditions, while the machining setup and measurement procedures were kept constant to ensure repeatability. No replicates were performed, as the full factorial design was considered sufficient to capture the effects of the process parameters. To minimize the influence of tool wear, the cutting insert was replaced after every six experimental runs (each cutting edge was used in three experiments), resulting in the use of five inserts in total. The experiments were conducted in a randomized order, ensuring that any wear effects were distributed across the design space. Moreover, all tests were performed under finishing conditions, and visual inspection confirmed the absence of significant tool degradation.

Regarding the *DE* values, they were derived by measuring the diameter of the specimens on the plane axes (X, Y), forming a cross. Then, the absolute mean value of the deviation between the ideal dimensions and the measured ones were calculated with Equation (1), where *DE* represents the dimensional error in μm. ${\bar{m}}_{a}$ is the mean value of the measured diameter, computed with Equation 2, whereas *n_a_* is the nominal dimension.

(1) $$DE=\left|{\bar{m}}_{a}-n_{a}\right|$$

(2) $${\bar{m}}_{a}=\frac{1}{2}\sum_{i=1}^{2}m_{a,i}$$

The machining of CFRP composites is inherently challenging due to their anisotropic and heterogeneous structure, which leads to non-uniform material removal, matrix smearing, and fiber–matrix debonding [23,24]. In contrast to conventionally manufactured composites, FDM-produced CFRP exhibits a layered architecture with weaker interlayer bonding, further influencing cutting mechanics and dimensional stability. Reported surface roughness values for polymer-based composites during finishing operations typically range between 1 μm and 4 μm, depending on cutting conditions and material structure. For example, Cococcetta et al. [25] reported surface roughness in the range of 2 μm to 10 μm for the edge milling of CFRP composites under various conditions. Srivastava and Mofakkirul [26] worked on the CFRP turning under similar cutting speeds and feeds. Their findings indicated a surface roughness close to 1.5 μm at depths of cut below 0.2 mm. Similar surface roughness values were reported by Bhushan et al. [27] for aluminum alloy composites as well. In the present study, the obtained *Ra* values (1.6 μm to 3.4 μm) fall within this range, indicating acceptable surface quality despite the inherent limitations of additively manufactured materials. However, the observed dimensional deviations highlight the sensitivity of such materials to process parameters, particularly depth of cut, due to their reduced stiffness and increased susceptibility to elastic recovery [28]. Analogous dimensional deviations are observed in the work of Muguthu and Gao for metal matrix composites [29].

**Figure 2:**
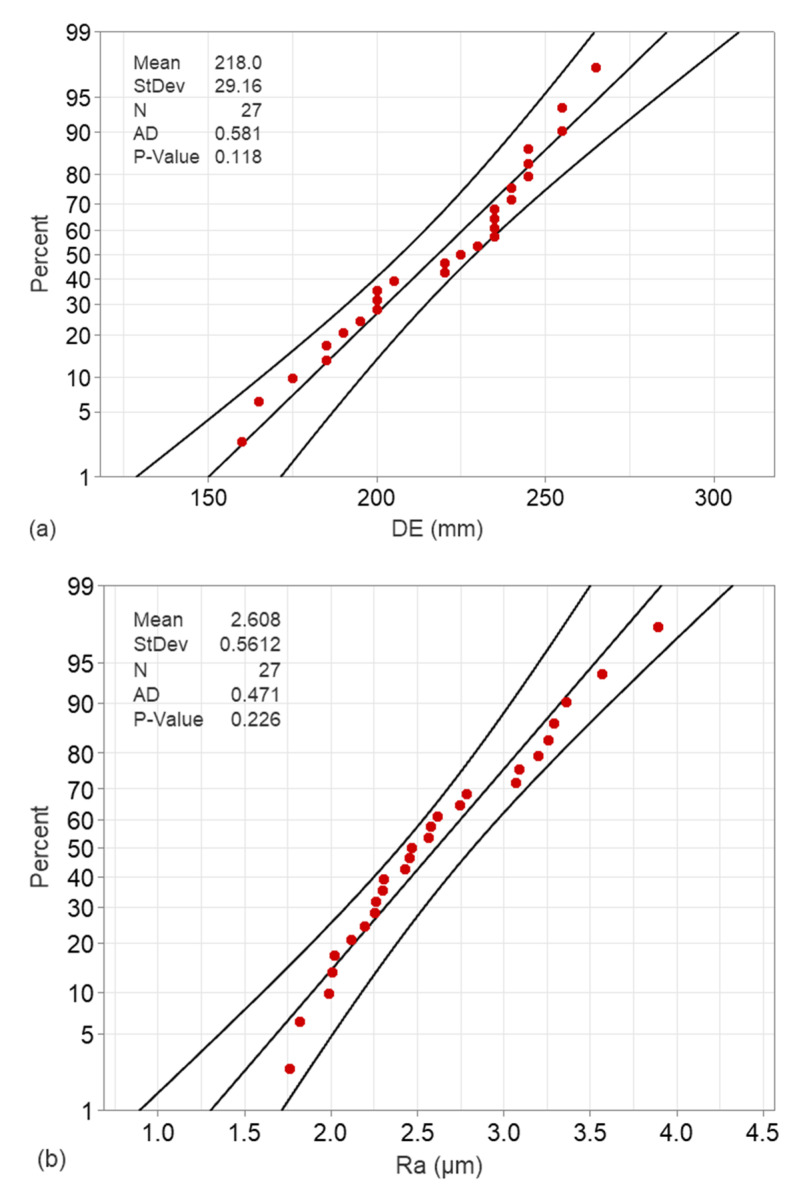
Probability plots of the experimental dimensional error (**a**) and the surface roughness (**b**).

The normality of the surface roughness and the dimensional error data were evaluated using the probability plots shown in Figure 2, constructed based on the Anderson–Darling test [30] with a 95% confidence level. The majority of the measured data points (red markers) exhibit close alignment with the reference straight line, indicating that both the *Ra* and the *DE* values conform satisfactorily to a normal distribution. Moreover, all points remain within the applied confidence bands (curved lines) and do not indicate significant departures from normality. Furthermore, the clustering of the data points around the fitted distribution line suggests uniform variance across the measurements, thereby supporting the validity of the normality assumption for subsequent statistical analysis. Finally, the quantitative results of the Anderson–Darling test further support these observations. For the dimensional error measurements (Figure 2a), the calculated *p*-value of 0.118 and Anderson–Darling statistic of 0.581 exceed the applied significance threshold, indicating no statistically significant deviation from normality at the 95% confidence level. Similarly, for the surface roughness data (Figure 2b), a higher *p*-value of 0.226 combined with a lower Anderson–Darling statistic of 0.471 confirms an even stronger agreement with the normal distribution. These results validate the applicability of parametric statistical methods and regression-based modeling for the analysis of both response variables.

### 2.3. Multi-Response Analysis

Multiple regression analysis is a widely applied statistical technique for describing and quantifying the relationship between a response variable and several independent variables. The method estimates the response by fitting a linear model that minimizes the residual error between experimental observations and predicted values. In machining-related investigations, multiple regression is commonly employed to evaluate the influence of process parameters such as cutting speed, feed rate, and depth of cut on surface roughness [31], cutting forces [32], cutting temperature [33] and other output variables. Equations (3) and (4) present the regression models obtained from the experimental dataset. Equation (3) describes the prediction of the dimensional error, whereas Equation (4) corresponds to the surface roughness prediction equation. Both models include linear, interaction, and quadratic terms to adequately capture the nonlinear behavior of the process. The regression equation for both *DE* and *Ra* were refined by using the backward elimination procedure [34], to eliminate any insignificant terms without sacrificing accuracy. Specifically, the predicted coefficient of determination was increased. Despite the fact that modern methods and techniques are available in the literature, such as Artificial Neural Networks (ANN) [35], the Adaptive Neuro Fuzzy Inference System (ANFIS) [36] and Support Vector Regression (SVR) [37], regression analysis is still used due to its simplicity and sufficient modeling power. Compared to the aforementioned methods, regression analysis does not require high end computing systems, neither large datasets for training.

(3) $$DE\left(\mu m\right)=86.7+0.692V_{c}-287.0f+96.9ap-0.001346{V_{c}}^{2}-23.21ap^{2}$$

(4) $$Ra\left(\mu m\right)=2.446-0.01097V_{c}-19.4f+1.285ap+0.000026V_{c}^{2}+186.7f^{2}-0.39ap^{2}+0.0367V_{c}\times f$$

To evaluate the statistical relevance of the model terms and quantify their individual contributions, an analysis of variance (ANOVA) was conducted on the response data. The ANOVA outcomes, including mean square values, *f*-statistics, and corresponding *p*-values, are summarized in Table 5 and Table 6 for the *DE* and *Ra* respectively. Model adequacy, variance explanation, and predictive capability were examined at a 95% confidence level. The calculated coefficients of determination for the *DE* model were determined as R^2^ = 96.35%, R^2^ (adjusted) = 95.48% and R^2^ (predicted) = 93.92%, indicating an excellent fit to the experimental data and satisfactory predictive performance, as a large proportion of the response variability is captured by the regression model. Statistical significance was assessed based on *p*-values, with terms exhibiting *p*-values lower than the significance level (α = 0.05) considered statistically meaningful. Nevertheless, statistical significance alone does not necessarily reflect the relative influence of each factor on the response. For this reason, the *f*-value defined as the ratio of the term mean square to the residual mean square was employed to evaluate the magnitude of each term’s effect. The analysis revealed that the depth of cut, together with the cutting speed, account for approximately 86% of the observed variation in the response. Since no replicated experiments were conducted, the error term in the ANOVA represents the residual error, incorporating both experimental variability and model inadequacy. Therefore, pure error could not be independently estimated.

In addition, the quality metrics of the second regression model are presented in Table 6. For this model, the coefficients of determination were calculated as R^2^ = 92.88%, R^2^ (adjusted) = 90.26% and R^2^ (predicted) = 85.99%. Although slightly lower than those of the first model, these values still demonstrate a strong agreement between the predicted and experimental results, as well as acceptable predictive reliability, confirming the suitability of the developed regression framework for describing the examined machining response. The three cutting parameters were able to explain 90% of the model’s variance.

Several studies in machining and manufacturing modeling have shown that, for limited datasets (typically less than 50 experiments), regression-based models can achieve prediction accuracies comparable to machine learning approaches, while maintaining significantly lower computational cost and higher interpretability. For instance, data-driven models such as ANN often require iterative training, architecture selection, and hyperparameter tuning, which increases computational effort and may introduce variability in the results. In contrast, second-order regression models involve the estimation of a small number of coefficients and can be obtained analytically with negligible computational cost. Indicatively, previous works in machining report similar prediction errors for ANN and RSM, which is comparable to the error levels obtained in the present study using regression. For example, a study in fiber bio-composite drilling [38] reported 0.16% for RSM and 0.09% for ANN. Similar observations have been reported in the broader literature, where RSM and ANN models yield comparable predictive performance for small experimental designs. A work on 4140 steel machining [32] revealed coefficients of determination for a variety of outputs between 0.89 and 0.97 with RSM, whereas with ANN, the range fluctuated between 0.95 and 0.99. Despite the fact that most of the time, ANN yields more accurate results, RSM provides additional advantages in terms of statistical significance analysis, factor contribution, and interaction interpretation.

**Figure 3:**
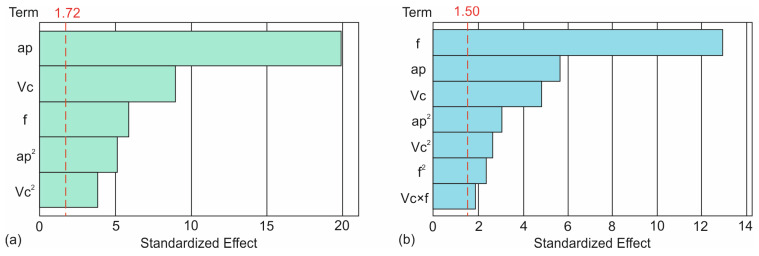
Pareto graphs for the contribution of the terms on the *DE* model (**a**) and the *Ra* model (**b**).

Figure 3 illustrates the contribution levels of each used term in the models. Specifically, Figure 3a highlights the aforementioned contribution to the *DE* model. The vertical dashed line at a standardized effect of 1.72 represents the statistical significance threshold at the selected confidence level. Therefore, terms exceeding this value are considered significant contributors to the response. Among all factors, depth of cut exhibits the dominant effect on dimensional error, showing a standardized effect substantially higher than all other terms. This confirms that dimensional stability in the turning of 3D-printed CFRP is primarily driven by the magnitude of material engagement, which directly affects the cutting forces and elastic recovery of the printed structure. The cutting speed appears to be the second most influential factor, indicating a meaningful, though secondary, contribution to dimensional error. This suggests that thermal and dynamic effects associated with higher speeds influence dimensional accuracy, but to a lesser degree than the mechanical loading imposed by depth of cut. The feed rate also exceeds the significance limit, indicating a statistically significant but comparatively smaller effect. Its influence is likely associated with changes in chip thickness and tool–workpiece interaction time, which moderately affect dimensional deviations. Regarding higher-order terms, *ap*^2^ is significant, confirming the non-linear relationship between depth of cut and dimensional error. This indicates that dimensional error does not increase linearly with *ap*, but accelerates beyond certain levels, which is consistent with the onset of increased tool deflection and compliance effects in finishing regimes. In contrast, *Vc*^2^ shows the lowest standardized effect among the listed terms and lies closer to the significance threshold, implying a weaker nonlinear contribution of cutting speed.

By observing Figure 3b, it is evident that feed rate is by far the most influential factor, exhibiting the largest standardized effect and clearly dominating all other terms. Its bar extends well beyond the reference significance threshold (red dashed line at 1.50), indicating that feed rate is the primary driver of the response variability in the model. Depth of cut ranks as the second most significant parameter. Although its contribution is substantially lower than that of feed rate, it still exceeds the significance limit by a wide margin, confirming its strong influence on the response. Cutting speed also shows a statistically significant effect, with a standardized effect clearly above the threshold. However, its influence is notably weaker compared to feed rate and depth of cut. Among the quadratic terms, *ap*^2^ and *Vc*^2^ exhibit moderate but still significant contributions, suggesting the presence of nonlinear effects associated primarily with depth of cut and cutting speed. In contrast, *f*^2^ has a smaller standardized effect, indicating that the non-linear contribution of feed rate is less pronounced compared to its dominant linear effect. The interaction term *Vc × f* lies close to the significance boundary and represents the least influential term included in the model. While its contribution is comparatively minor, the fact that it is close to the threshold suggests a weak but non-negligible interaction between cutting speed and feed rate. Overall, the Pareto analysis confirms that the linear effects of feed rate and depth of cut are the primary factors, followed by cutting speed, while quadratic and interaction terms play a secondary role. This hierarchy of effects is consistent with the ANOVA results and supports the physical interpretation of the machining process, where feed rate primarily controls material engagement and surface formation mechanisms.

## 3. Results and Discussion

### 3.1. Analysis of the Parameters’ Influence on the Generated Responses

**Figure 4:**
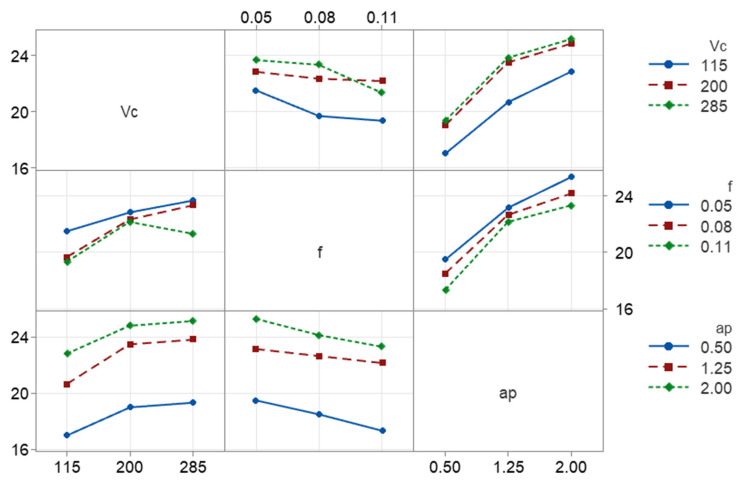
The interaction plots for the experimental responses with respect to the cutting parameters: *DE*.

To analyze the combined influence of the parameters, interaction plots were plotted. Figure 4 corresponds to the interaction plot for the dimensional error. Across most parameter combinations, dimensional error tends to increase with increasing cutting speed, particularly at higher depths of cut. However, the slopes are not perfectly parallel, indicating interaction effects. At a low depth of cut (*ap* = 0.5 mm), the increase in *DE* with *Vc* is moderate. At higher depths (*ap* = 1.25 and 2.0 mm), the rise in *DE* with *Vc* becomes more evident. This suggests that the influence of cutting speed is amplified when the material engagement is larger, likely due to increased cutting forces and thermal effects interacting with the structural compliance of the CFRP workpiece. Continuing, feed rate shows a noticeable influence on dimensional error, although weaker than depth of cut. Despite being weaker, the effect of feed rate on dimensional error may be associated with the layered architecture of the FDM material. At very low feeds, increased tool–material interaction promotes plowing and elastic recovery [39], while moderate feed rates favor more efficient material removal. Additionally, the anisotropic filament structure may lead to direction-dependent deformation behavior [40], contributing to the observed trend. At medium *ap*, increasing feed causes a moderate decrease in *DE*. At higher or lower *ap*, the increase becomes steeper. The non-parallel lines in the *Vc*–*f* and *f*–*ap* subplots indicate that feed does not act independently. Instead, its effect becomes stronger when combined with higher depth of cut, suggesting a coupled mechanical loading effect. Depth of cut clearly produces the largest change in dimensional error. Across all cutting speeds and feeds, *DE* increases significantly as *ap* shifts from 0.5 mm to 2.0 mm. The slope of the *ap* curves is steep and consistent. Higher speeds further magnify this increase. To summarize, the interaction plot reinforces that dimensional error in the dry turning of 3D-printed CFRP is primarily controlled by depth of cut, with cutting speed and feed acting as secondary modifiers.

**Figure 5:**
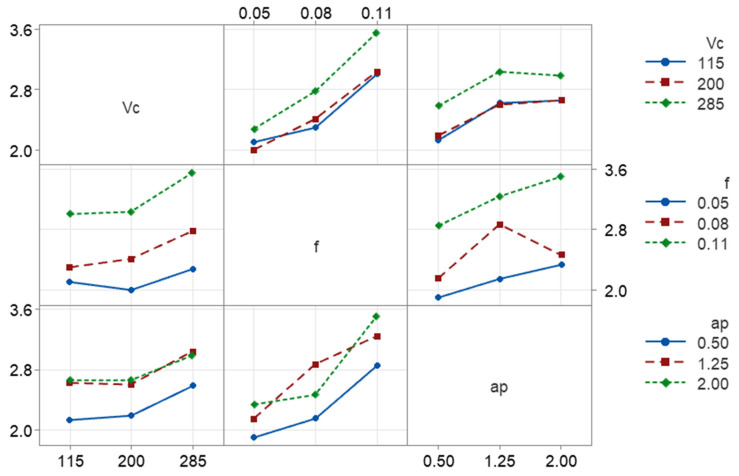
The interaction plots for the experimental responses with respect to the cutting parameters: *Ra*.

The interaction plot of Figure 5 illustrates the combined effects of cutting speed, feed, and depth of cut on surface roughness and reveals that feed rate is the dominant controlling factor, while cutting speed and depth of cut mainly influence *Ra* through secondary and interaction effects. Surface roughness shows a moderate dependence on cutting speed. In most combinations of feed and depth of cut, *Ra* increases as *Vc* increases from 115 m/min to 285 m/min. The non-parallel trends seen across different feed levels indicate a noticeable interaction between cutting speed and feed, suggesting that the influence of *Vc* on *Ra* becomes stronger at higher feed rates. Nevertheless, *Vc* alone does not dominate the roughness response. Feed rate exhibits a strong and consistent effect on surface roughness. *Ra* increases significantly as feed rises from 0.05 mm/rev to 0.11 mm/rev across all cutting speeds and depths of cut. The steep slopes confirm that feed is the primary contributor to surface roughness. The presence of non-parallel lines further indicates interaction effects between feed and both *Vc* and *ap*, amplifying roughness at higher parameter combinations. Depth of cut has a secondary but non-negligible effect on *Ra*. Surface roughness generally increases with *ap*, although the effect is weaker than that of feed. The trends suggest that *ap* mainly acts by modulating the feed-induced roughness, rather than independently controlling *Ra*. Mild curvature and divergence among the lines imply limited interaction between *ap* and the remaining parameters. In summary, the interaction plot confirms that feed rate directly controls surface roughness, with cutting speed and depth of cut contributing through weaker main effects and interaction terms. These observations are consistent with the ANOVA results, where feed-related terms are expected to dominate the roughness response.

**Figure 6:**
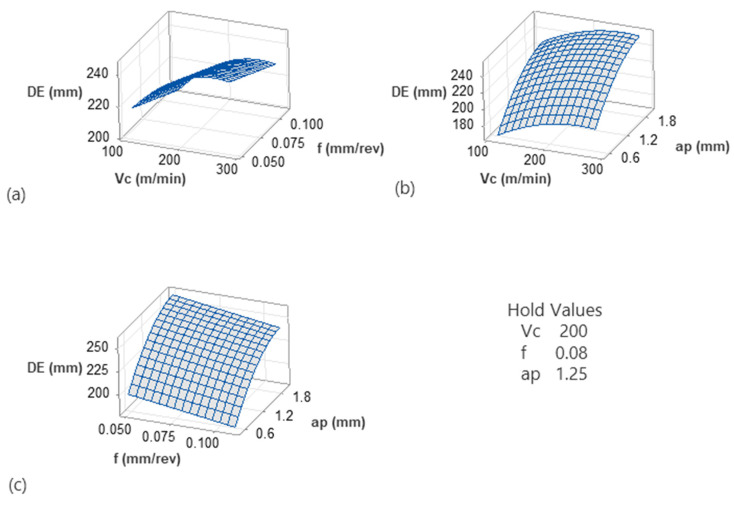
The 3D surface plots for the *DE* model: *Vc*-*f* (**a**), *Vc*-*ap* (**b**) and *f*-*ap* (**c**).

**Figure 7:**
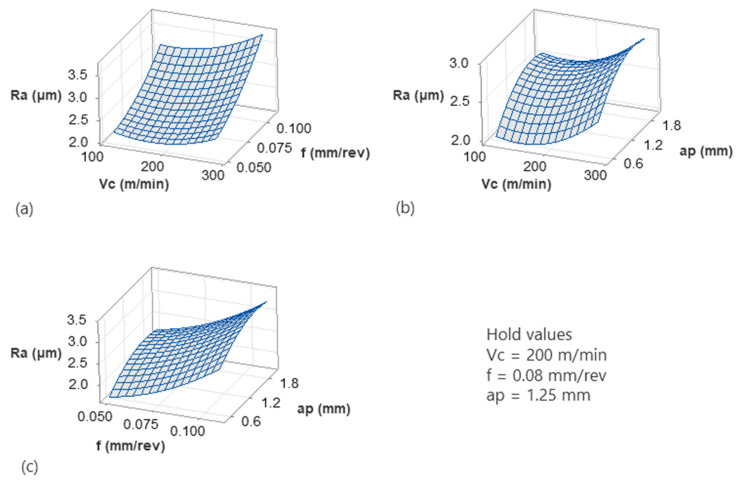
The 3D surface plots for the *Ra* model: *Vc*-*f* (**a**), *Vc*-*ap* (**b**) and *f*-*ap* (**c**).

Further processing of the models revealed a more complex behavior of the specific material when machined. Figure 6 and Figure 7 illustrate the 3D surface plots of the responses against the three inputs in a pairwise manner. Specifically, Figure 6a depicts the interactions of cutting speed with feed. It is shown that dimensional error decreases moderately with feed, whereas lower speeds contribute towards reduced dimensional accuracy. Similar findings regarding the effect of speed were shown in the work of Njuguna Muguthu and Gao [29] for metal matrix composites. Continuing, the surface shows a gradual upward slope along the *Vc* axis, indicating that higher speeds slightly increase *DE*. The effect of feed is comparatively weaker but still noticeable. The slight curvature of the surface suggests mild interaction between *Vc* and *f*. Overall, this surface indicates that while both parameters influence *DE*, their combined effect is secondary compared to depth of cut. Figure 6b shows a much steeper surface along the *ap* direction. Dimensional error increases significantly as depth of cut increases from 0.5 mm to 2.0 mm as already discussed in the corresponding interaction plot. A work on the dry turning of AISI 4340 [41] revealed a contradictory finding regarding feed, compared to this study, probably due to the structure of the material leading to different thermal patterns. However, aligned with the present study, depth of cut was identified as a factor with strong influence on the dimensional accuracy. This finding is possibly linked to the fact that depth of cut is directly related to the amount of material removed and not to the nature of the material. Additionally, the curvature of the surface confirms the non-linear contribution of *ap*^2^ identified in the regression model and Pareto analysis. The increase in *DE* becomes more visible at higher *ap* values, indicating accelerating dimensional deviation beyond moderate engagement levels. The cutting speed further amplifies this effect, particularly at deeper cuts, highlighting a *Vc* × *ap* interaction. This suggests that higher cutting speeds intensify force-related deflections when material engagement is large. In the plot of Figure 6c, depth of cut again shows the dominant slope, reinforcing its primary role in controlling dimensional error. Feed acts decreasingly to *DE*, but mainly when combined with lower *ap* values. However, its magnitude is small compared to other factors. Overall interpretation of the surface plots indicates that depth of cut is the primary parameter affecting dimensional error. This finding was already reported and validated with microscopy by a previous study [17] of the authors for 3D-printed CFRP machining. The presence of curvature confirms the significant quadratic term (*ap*^2^). Finally, cutting speed and feed rate exert secondary influences. From a machining perspective, dimensional stability is primarily controlled by limiting material engagement (*ap*), while cutting speed and feed should be selected to avoid amplifying force-induced deflection. These graphical observations align with the Pareto analysis, interaction plots, and statistical validation results, providing coherent support for the developed regression model.

The observed trend in Figure 7a indicates that *Ra* increases strongly with feed rate, whereas *Vc* has a weaker, non-linear effect. Specifically, at low feeds, increasing *Vc* slightly reduces *Ra*. On the other hand, at higher feeds, *Ra* increases again with *Vc*. This finding suggests that feed rate dominates surface roughness due to geometric effects (feed marks and uncut chip thickness). Similar findings for the effect of speed and feed were reported in several studies for normal CFRP materials during drilling [42] and milling [43,44]. The effect of feed is similar to metallic materials as well during turning [35]. Moreover, the curvature along *Vc* suggests competing mechanisms. While moderate *Vc* provides better shearing and therefore a smoother surface, high *Vc* tends to increase vibrations, thermal softening and probably material smearing. Continuing with Figure 7b, it is evident that *Ra* increases with depth of cut. It is shown that *Ra* slightly decreases at higher speeds and increases again at intermediate speed values, while it remains low at the lowest depth of cut value. Overall, depth of cut has a clear degrading effect on surface quality, especially at high cutting speeds, possibly due to higher cutting forces, tool deflection and dynamic instability. The *Vc* curvature again points to a non-monotonic speed effect, often linked to chatter onset and thermal–mechanical transitions. Finally, the interaction between *f* and *ap* acts increasingly, as shown in Figure 7c. However, the slope along *f* is steeper than along *ap*, suggesting that feed rate controls surface geometry directly. Overall, their combination is critical as aggressive cutting conditions, i.e., deeper cuts and higher feeds, compound roughness.

### 3.2. Evolutionary Optimization of the Cutting Conditions for Minimizng Responses

**Figure 8:**
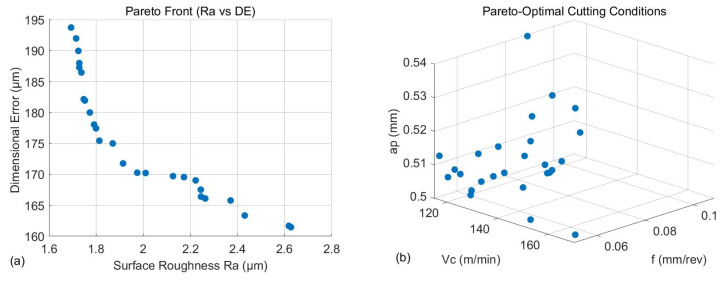
The optimization plots: Pareto front (**a**) and the solutions (**b**).

To optimize the machining process, both *DE* and *Ra* were considered, since in light and finishing turning operations, the final accuracy results from a balance between dimensional errors and surface quality. To address this multi-objective problem, the Non-Dominated Sorting Genetic Algorithm II (NSGA-II) was adopted, as it is a well-established optimization technique for handling multiple objectives [37,45]. The algorithm follows a population-based strategy and is capable of identifying a set of Pareto-optimal solutions within a single optimization run. Solution ranking is performed through non-dominated sorting, while population diversity is preserved using a crowding distance criterion. Genetic operators such as crossover and mutation are applied to enhance both exploration and convergence of the solution space. Owing to its fast sorting procedure, elitist strategy, and reduced computational complexity, NSGA-II offers high efficiency and scalability. For the optimization problem examined in this study, involving three continuous decision variables, the population size was set to 200 and the maximum number of generations to 50, imposing termination criteria when the maximum generations are reached. The optimization results are illustrated in Figure 8, where Figure 8a illustrates the Pareto front and Figure 8b the corresponding combinations of cutting parameters that yielded the Pareto-optimal solutions. The optimal solutions are dispersed in a large cluster between 120 m/min and 160 m/min cutting speed, at depths below 0.52 mm and at feeds ranging from 0.05 mm/rev to 0.10 mm/rev. Although no formal uncertainty quantification was performed, the robustness of the optimization results is supported by the high predictive accuracy of the regression models and the low validation errors. Additionally, the clustering of Pareto-optimal solutions within a narrow parameter range indicates the existence of a stable optimal region, suggesting limited sensitivity to model uncertainty.

### 3.3. Validation of the Models’ Performance

**Figure 9:**
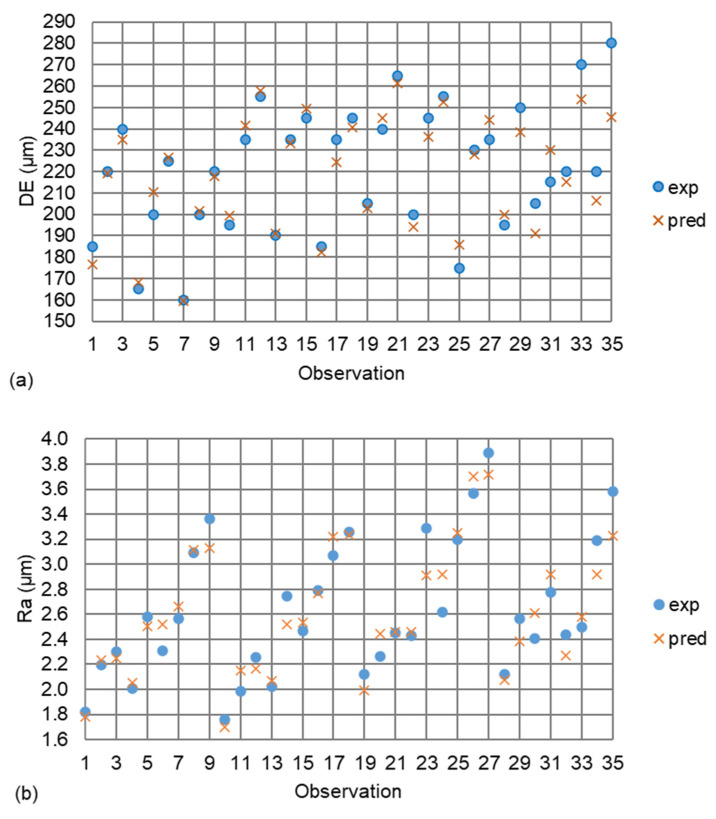
Comparison plots for the experimental vs. the predicted responses: *DE* (**a**) and *Ra* (**b**).

For model validation, an independent experimental set was defined using intermediate cutting conditions located between the factorial levels. This approach ensures that the predictive capability of the model is evaluated under unseen operating conditions, thus providing a more rigorous assessment of its generalization performance. Table 7 contains the parameter combinations of the extra experiments, as well as the corresponding results. By observing Table 7, it is evident that both models yielded accurate results, proving their robustness. Specifically, the relative error between the experimental and the predicted values of the *DE* was computed between −12.4% and 7.0%, whereas for the *Ra*, it was calculated between −9.8% and 8.2%. To visualize the total 35 observations (27 initial tests and 8 validation tests), Figure 9 was employed. Specifically, Figure 9a illustrates the comparison between the experimental and the predicted values of *DE*, whereas Figure 9b compares the equivalent data points for *Ra*. By observing the graphs, a strong correlation is evident. The mean absolute percentage error (MAPE) was computed equal to 3% and 4.8% for *DE* and *Ra*, respectively.

To further assess the predictive capability of the regression model, a k-fold cross-validation [46,47] procedure was applied. The dataset (27 experiments) was randomly divided into five subsets. In each iteration, four subsets (≈80% of the data) were used for validation while the remaining subset was used for testing. This process was repeated five times so that each data point was predicted once as unseen data. The prediction errors from all folds were then aggregated to compute Prediction Residual Error Sum of Squares (PRESS), Root Mean Square Error (RMSE), and Mean Absolute Error (MAE). This approach provides an objective estimate of the model’s generalization ability and reduces the risk of overfitting. The obtained metrics are summarized in Table 8.

To compute the metrics of Table 8, Equations (5)–(7) were used accordingly, with *y_exp_* being the experimental value of the responses and *y_pred_* being the predicted one. *PRESS* is essentially the sum of all squared residuals from the folds. *RMSE* is the square root of the average of the squared residuals. Finally, *MAE* is the average of the absolute residual values.

(5) $$PRESS={\sum_{i=1}^{27}\left(y_{\exp,i}-y_{pred,i}\right)}^{2}$$

(6) $$RMSE=\sqrt{\frac{1}{27}\sum {\left(y_{\exp}-y_{pred}\right)}^{2}}$$

(7) $$MAE=\frac{1}{27}\sum \left|y_{\exp}-y_{pred}\right|$$

The error magnitudes are very small relative to the overall *DE* range (150 μm to 250 μm), indicating high predictive precision. The same applies for the *Ra* model, with the error metrics *RMSE* and *MAE* being approximately equal to 0.15 μm and 0.11 μm respectively. The corresponding *PRESS* values (807.87 for *DE* and 0.5830 for *Ra*) are consistent with the scale of each response and should be interpreted in conjunction with the error metrics. Overall, the low prediction errors and consistent cross-validation performance demonstrate the robustness and generalization capability of the proposed regression models.

## 4. Conclusions

This study investigated the influence of cutting parameters on surface roughness and dimensional error during the turning of additively manufactured PET-G-based CFRP specimens. The results demonstrated that depth of cut is the dominant factor affecting dimensional error, contributing approximately 72% of the total variation, followed by cutting speed and feed rate. In contrast, surface roughness is primarily governed by feed rate, which accounts for approximately 68% of the variability, confirming its critical role in surface generation during machining. The observed trends indicate that increased depth of cut and feed rate lead to higher dimensional deviations, likely due to increased cutting forces and tool deflection, while higher feed rates significantly deteriorate surface finish. Cutting speed exhibited a secondary but non-negligible influence on both responses. Multi-objective optimization revealed that optimal machining performance is achieved at moderate cutting speeds (120–160 m/min), low depths of cut (<0.52 mm), and low-to-medium feed rates (0.05–0.10 mm/rev), enabling a balanced trade-off between dimensional accuracy and surface quality. The developed response surface models demonstrated strong predictive capability, with MAPE values of 3% for dimensional error and 4.8% for surface roughness, confirming their suitability for process prediction and optimization. Overall, the study revealed that limiting depth of cut and feed rate is essential for improving machining performance in 3D-printed CFRP materials, providing practical guidelines for achieving enhanced dimensional accuracy and surface integrity.

## 5. Limitations and Future Work

The present investigation does not incorporate dynamic process variables, such as vibration signals, chatter effects, tool deflection and temperature effects. Consequently, the developed models are based exclusively on continuous input parameters and are inherently unable to capture abrupt changes in machining states. In view of this limitation, future research will focus on the integration of sensor-based monitoring systems capable of recording acceleration and temperature signals, with the objective of developing a dynamic prediction framework with enhanced predictive accuracy. The incorporation of suitable sensors and data acquisition architectures is expected to enable real-time characterization of the machining process. Moreover, such a dynamic modeling approach may serve as a foundational step toward the implementation of a digital twin–based monitoring and control system.

## Figures and Tables

**Table 1:** Composition and specifications of the CFRP filament.

Specification	Value
Density	1190 kg/m^3^
Elastic modulus	3.8 Gpa (at 1 mm/min)
Yield strength	52.5 Mpa (at 50 mm/min)
Yield strain	4.2% (at 50 mm/min)
Strain at break	8% (at 50 mm/min)
Heat distortion temperature	80 °C
Base material	80% PET-G
Reinforcement material	20% carbon fibers

**Table 2:** 3D printer settings for the CFRP workpieces.

Printer Setting	Value
Nozzle temperature	255 °C
Bed temperature	70 °C
Layer thickness	0.2 mm
Printing speed	40 mm/s
Infill density	50%
Infill pattern	Rectilinear

**Table 3:** The predictor levels of the analysis.

Level	*Vc* (m·min^−1^)	*f* (mm·rev^−1^)	*ap* (mm)
+1	285	0.11	2.00
0	200	0.08	1.25
−1	115	0.05	0.50

**Table 4:** The design of experiments and the measured responses.

Test	Run Order	*Vc* (m·min^−1^)	*f* (mm·rev^−1^)	*ap* (mm)	*DE* (μm)	*Ra* (μm)
1	26	115	0.05	0.50	185	1.823
2	10	115	0.05	1.25	220	2.198
3	14	115	0.05	2.00	240	2.301
4	25	115	0.08	0.50	165	2.009
5	3	115	0.08	1.25	200	2.580
6	5	115	0.08	2.00	225	2.308
7	21	115	0.11	0.50	160	2.566
8	22	115	0.11	1.25	200	3.090
9	16	115	0.11	2.00	220	3.362
10	6	200	0.05	0.50	195	1.762
11	19	200	0.05	1.25	235	1.989
12	2	200	0.05	2.00	255	2.253
13	9	200	0.08	0.50	190	2.020
14	13	200	0.08	1.25	235	2.744
15	4	200	0.08	2.00	245	2.466
16	17	200	0.11	0.50	185	2.788
17	18	200	0.11	1.25	235	3.067
18	15	200	0.11	2.00	245	3.255
19	24	285	0.05	0.50	205	2.121
20	20	285	0.05	1.25	240	2.262
21	8	285	0.05	2.00	265	2.453
22	7	285	0.08	0.50	200	2.429
23	12	285	0.08	1.25	245	3.288
24	27	285	0.08	2.00	255	2.616
25	11	285	0.11	0.50	175	3.199
26	23	285	0.11	1.25	230	3.566
27	1	285	0.11	2.00	235	3.890

**Table 5:** ANOVA results for the dimensional error model.

Source	Degree of Freedom	Sum of Squares	Mean Square	*f*-Value	*p*-Value	Contribution %
Model	5	21305.1	4261	110.76	0.000	
Error	21	807.9	38.5			
Total	26	22113				
R-sq = 96.35% R-sq (adj) = 95.48% R-sq (pred) = 93.92%						
Term						
*Vc*	1	3068.1	3068.1	79.75	0.000	14.40
*f*	1	1334.7	1334.7	34.7	0.000	6.27
*ap*	1	153.125	153.125	398.04	0.000	71.87
*Vc^2^*	1	567.1	567.1	14.74	0.001	2.66
*ap^2^*	1	1022.7	1022.7	26.58	0.000	4.80

**Table 6:** ANOVA results for the surface roughness model.

Source	Degree of Freedom	Sum of Squares	Mean Square	*f*-Value	*p*-Value	Contribution %
Model	7	7.6049	1.08642	35.42	0.000	
Error	19	0.5828	0.03068			
Total	26	8.1877				
R-sq = 92.88% R-sq (adj) = 90.26% R-sq (pred) = 85.99%						
Term						
*Vc*	1	0.71481	0.71481	23.3	0.000	9.40
*f*	1	5.14242	5.14242	167.64	0.000	67.62
*ap*	1	0.97394	0.97394	31.75	0.000	12.81
*Vc^2^*	1	0.21069	0.21069	6.87	0.017	2.77
*f^2^*	1	0.16946	0.16946	5.52	0.030	2.23
*ap^2^*	1	0.2885	0.2885	9.4	0.006	3.79
*Vc × f*	1	0.10509	0.10509	3.43	0.080	1.38

**Table 7:** Comparison between the validation experimental results and the predicted ones.

Test No	*Vc* (m·min^−1^)	*f* (mm·rev^−1^)	*ap* (mm)	*DE_, exp_* (μm)	*DE_, pred_* (μm)	Relative Error (%)	*R_a, exp_* (μm)	*R_a, pred_* (μm)	Relative Error (%)
1	150	0.07	0.75	195	199.7	2.4	2.122	2.072	−2.4
2	150	0.07	1.75	250	238.6	−4.6	2.567	2.382	−7.2
3	150	0.10	0.75	205	191.1	−6.8	2.409	2.607	8.2
4	150	0.10	1.75	215	230.0	7.0	2.778	2.917	5.0
5	250	0.07	0.75	220	215.1	−2.2	2.440	2.272	−6.9
6	250	0.07	1.75	270	254.0	−5.9	2.501	2.582	3.2
7	250	0.10	0.75	220	206.5	−6.1	3.192	2.917	−8.6
8	250	0.10	1.75	280	245.4	−12.4	3.578	3.227	−9.8

**Table 8:** k-fold cross-validation metrics.

	DE Model	Ra Model
PRESS	807.87	0.5830
RMSE	5.47 μm	0.1469 μm
MAE	4.50 μm	0.1131 μm

## Data Availability

Data are contained within the article.
